# Developmental literacy and epistemic dignity as primary child protection: a developmentally staged, disability-inclusive public-health framework for preventing adverse childhood experiences

**DOI:** 10.3389/fpubh.2026.1862974

**Published:** 2026-06-10

**Authors:** Eik Niederlohmann

**Affiliations:** Kliniken Erlabrunn, Department of Psychosomatic Medicine and Psychotherapy, Breitenbrunn, Germany

**Keywords:** adverse childhood experiences, developmental literacy, epistemic dignity, health-promoting schools, implementation science, neurodiversity, nurturing care, primary child protection

## Abstract

Adverse childhood experiences (ACEs), toxic stress, relational insecurity, and structural adversity are major public-health concerns for children and adolescents, but ACE evidence should not be treated as an individual diagnostic score or deterministic prognosis. This Hypothesis and Theory article proposes developmental literacy and epistemic dignity as linked upstream constructs for primary child protection. Developmental literacy is defined as developmentally staged and disability-inclusive neurobiopsychosocial knowledge and skills concerning bodies, emotions, stress, attachment, co-regulation, play, nutrition, safety, rights, boundaries, non-violent care, and help-seeking. Epistemic dignity refers to the supported capacity and right of children—including preverbal, disabled, neurodivergent, and communication-diverse children—to have bodily, emotional, relational, play-based, and communicative signals interpreted with seriousness, humility, and appropriate response. Drawing on research on ACEs and positive childhood experiences (PCEs), nurturing care, relational health, health-promoting schools, social and emotional learning, mental-health literacy, sexuality/safety education, child participation, epistemic injustice, disability studies, implementation science, and critiques of ACE and trauma-informed practice, the article develops a universal, proportionate, relationally safe, anti-bias, and non-coercive public-health framework. It argues that early-childhood services, schools, pediatric/public-health touchpoints, caregiver support, and community systems can provide age-appropriate developmental knowledge without turning schools into clinics, teachers into trauma detectors, or children into individual risk scores. The framework does not recommend routine individual ACE-score screening in schools as a default practice; it supports ethically governed, service-linked pathways for recognizing distress and unmet support needs. Coercive reproductive control is treated only as a rejected comparator because it targets reproductive status rather than modifiable developmental environments, support access, and institutional trust. The article concludes with safeguards and testable hypotheses for evaluating developmental literacy, epistemic dignity, relational safety, accessibility, help-seeking, response quality, caregiver support, referral continuity, and unintended harms.

## 1 Introduction

Adverse childhood experiences (ACEs) are important population-level indicators of preventable developmental adversity and later mental, physical, educational, and social burden. The original ACE study and subsequent systematic reviews document graded associations between cumulative adversity and depression, suicidality, substance use, cardiovascular disease, metabolic disease, and other major outcomes (1–3). Surveillance data also show that adversity is common and socially patterned, with substantial proportions of adults and adolescents reporting one or more ACEs (4, 5). These data make ACEs important for child public health, but they do not by themselves define a humane, developmentally precise, or ethically safe prevention strategy.

Developmental science adds an important qualification. Children do not merely remember adversity; they develop within relational, biological, educational, and institutional conditions that can amplify or buffer stress. Repeated threat, humiliation, neglect, violence, instability, or chronic caregiver distress may contribute to altered stress physiology, affect regulation, learning, body awareness, and health risk, especially when buffering relationships are absent (6, 7). Yet developmental pathways are not linear or inevitable. Multifinality, equifinality, resilience, timing, cultural context, disability, service access, socioeconomic conditions, and protective relationships all shape outcomes (8–19). The prevention task is therefore not to label individual children as damaged, but to improve the developmental environments in which risk and protection are produced.

Many child-protection and child-mental-health systems remain strongest after harm has become visible: after maltreatment, psychiatric decompensation, school failure, family crisis, or statutory intervention. Downstream intervention is indispensable, but it is insufficient if ACEs, toxic stress, relational insecurity, and social determinants are major drivers of child-health inequity. Primary child protection should therefore ask not only how to respond during crisis, but how to make preventable developmental harm less likely in the first place (20–25).

This manuscript proposes that two linked constructs can help integrate currently fragmented literatures and practices: developmental literacy and epistemic dignity. Developmental literacy refers to developmentally staged and disability-inclusive neurobiopsychosocial knowledge and skills concerning bodies, emotions, stress, attachment, co-regulation, play, nutrition, safety, rights, boundaries, and help-seeking. Epistemic dignity refers to the supported capacity and right of children to have their bodily, emotional, relational, and communicative signals interpreted with seriousness, humility, and appropriate response. Together, these constructs support a form of primary child protection that is universal, proportionate, relationally safe, and non-coercive.

The article has five aims. First, it clarifies how ACEs are used in this framework as population-level indicators rather than individual diagnostic scores. Second, it operationalizes developmental literacy and epistemic dignity and differentiates them from adjacent constructs. Third, it explains how age-appropriate neurobiopsychosocial content can be integrated into early-childhood and school health education without turning education into psychotherapy. Fourth, it strengthens the model through developmental specificity, anti-ableist and anti-bias design, and trauma-informed caution. Fifth, it outlines testable hypotheses and implementation safeguards for future public-health research.

## 2 Framework-development approach

This is a Hypothesis and Theory article and a theory-generating narrative synthesis, not a systematic review. Sources were selected purposively to build and bound a testable framework for upstream child protection. Selection prioritized: (a) flagship epidemiological and public-health sources on ACEs, PCEs, violence prevention, and social determinants; (b) developmental and early-childhood sources on toxic stress, relational health, nurturing care, co-regulation, play, nutrition, and caregiver support; (c) child-rights and epistemic-injustice literature relevant to children as knowledge subjects; (d) school health, social and emotional learning, health literacy, mental-health literacy, sexuality education, and safety education; (e) disability, neurodiversity, Universal Design, early intervention/ECSE, anti-ableist, and anti-bias implementation; (f) implementation science; and (g) critical literature on ACE use, trauma-informed practice, screening, and unintended harms (1–59).

The literature was included when it helped answer one of four framework questions: which developmental mechanisms plausibly generate avoidable harm; which public-health levers operate at a modifiable causal level; how prevention can be delivered through universal and non-stigmatizing infrastructure; and which propositions are specific enough to be tested. Sources were excluded when they were primarily clinical treatment manuals without public-health relevance, purely national policy commentary without wider transferability, or risk-category approaches inconsistent with the manuscript’s anti-coercive and anti-stigmatizing logic.

This approach has limitations. The synthesis is not exhaustive, may underrepresent non-English and non-Western literature, and does not estimate intervention effectiveness. Its purpose is to integrate a dispersed evidence base into a conceptually coherent and empirically testable model. Because the model addresses children across developmental stages, it requires further adaptation and testing across cultures, school systems, early-childhood services, disability communities, safeguarding laws, and health-system contexts.

## 3 Use and limits of ACEs in this framework

The manuscript uses ACEs as population-level signals of preventable developmental adversity, not as deterministic predictors of individual outcomes and not as diagnostic labels for children, caregivers, or families. ACE exposure is associated with elevated risk at the group level, but individual trajectories remain heterogeneous. ACE counts also do not capture timing, severity, chronicity, meaning, protective relationships, disability-related barriers, structural inequity, or service access. Contemporary critiques therefore warn against cumulative-risk simplification, trauma determinism, and the use of ACE scores as individual prediction tools (8, 9, 26, 27).

This distinction is central to the framework. ACE research helps justify upstream prevention because it reveals patterns of exposure and burden. It does not justify treating individual children as forecastable risk cases or treating families as fixed risk types. Baldwin and colleagues showed that ACE scores can forecast mean group differences but have poor accuracy for identifying individuals at high risk for later health problems (9). Finkelhor and Loveday and colleagues likewise caution that ACE screening raises questions of benefit, harm, response capacity, and ethical implementation (26, 27).

Accordingly, this manuscript does not recommend routine semiannual ACE-score screening of individual pupils as a default school practice. Periodic population monitoring of school climate, connectedness, violence exposure, food insecurity, help-seeking barriers, or service accessibility may be useful when ethically governed and linked to action. However, individual ACE-score collection in schools risks stigma, surveillance, disclosure without support, data misuse, false reassurance, false classification, and teacher overburden if not embedded in a robust safeguarding and care system. Any structured identification process should be voluntary or assent-based where appropriate, developmentally and disability accessible, confidential within clear safeguarding limits, minimally intrusive, culturally sensitive, professionally supervised, and evaluated for unintended harms.

The framework therefore shifts the question from “Which child has a high ACE score?” to “Which developmental environments, institutional responses, and help-seeking pathways make preventable harm more or less likely?” This preserves the public-health value of ACE research while avoiding deterministic, individualized, or coercive applications.

## 4 Operational definitions and conceptual differentiation

**Figure 1:**
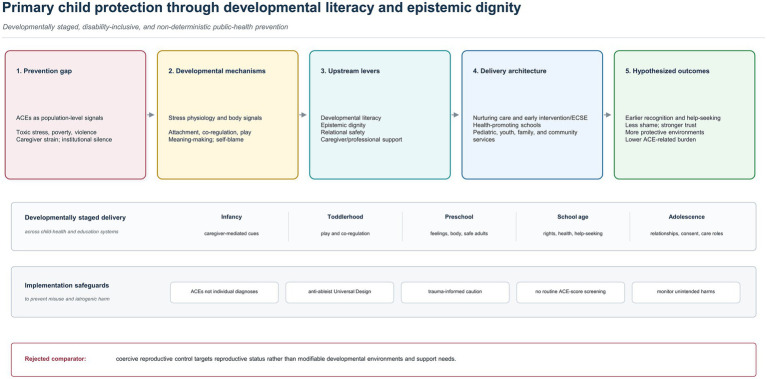
Infographic titled “Primary child protection through developmental literacy and epistemic dignity.” It shows five left-to-right panels: prevention gap, developmental mechanisms, upstream levers, delivery architecture, and hypothesized outcomes. ACEs, toxic stress, poverty, violence, caregiver strain, and institutional silence lead to mechanisms including stress physiology, attachment, co-regulation, play, meaning-making, and self-blame. Levers are developmental literacy, epistemic dignity, relational safety, and caregiver/professional support. Delivery is staged from infancy to adolescence across early intervention, health-promoting schools, pediatric, youth, family, and community services. Safeguards include non-diagnostic ACE use, Universal Design, trauma-informed caution, no routine ACE-score screening, harm monitoring, and rejection of coercive reproductive control.

Figure 1 summarizes the proposed pathway, and Table 1 operationalizes the core constructs. Developmental literacy is broader than general health literacy because it focuses on development, relationships, stress, meaning-making, rights, safety, and service pathways (58, 59). It overlaps with mental-health literacy but does not reduce children’s experience to disorder recognition (29, 30). It overlaps with social and emotional learning but adds body knowledge, child protection, adversity, help-seeking, caregiver support, and service linkage (25, 28). Epistemic dignity overlaps with participation rights but emphasizes the credibility, interpretability, and institutional reception of children’s signals (34–38).

**Table 1:** Operational definitions and differentiation from adjacent constructs.

Construct	Operational definition	Main prevention function	Differentiation and safeguards
Developmental literacy	Developmentally staged and disability-inclusive neurobiopsychosocial knowledge and skills concerning bodies, emotions, stress, attachment, co-regulation, play, nutrition, safety, rights, boundaries, and help-seeking.	Creates shared, usable language for children, caregivers, educators, clinicians, and services before crisis.	Broader than health literacy; more developmental than mental-health literacy; more safety and service-linked than SEL. Does not make children responsible for changing unsafe environments.
Epistemic dignity	The supported capacity and right of children, including preverbal, disabled, neurodivergent, and communication-diverse children, to have bodily, emotional, relational, and communicative signals interpreted with seriousness and humility.	Reduces hermeneutical isolation, testimonial dismissal, ridicule, and institutional silence.	Extends participation rights by emphasizing credibility, interpretive resources, responsible hearing, and actual response. Does not assume adult-like autonomy at all ages.
Relational safety	Interactional and institutional conditions under which prevention is experienced as usable rather than shaming, intrusive, coercive, or futile.	Increases trust, disclosure safety, caregiver engagement, and service uptake.	More specific than benevolent intent or generic trauma-informed language. Requires monitoring of shame, mistrust, disclosure without support, and teacher burden.
Primary child protection	Universal and proportionate prevention that reduces modifiable developmental harm before crisis entry into statutory child protection, specialist care, or emergency services.	Moves child protection upstream while keeping statutory response available when needed.	Not a substitute for child-protection law, pediatric care, psychotherapy, social services, poverty reduction, or specialist disability support.
Neurobiopsychosocial developmental education	Age and context-appropriate educational content about brain–body stress, emotions, relationships, care, health, nutrition, rights, violence prevention, consent, and help-seeking.	Provides external concepts that help children and adults distinguish normal developmental variation from harmful relational or institutional patterns.	Not a clinical trauma curriculum; not universal psychotherapy; not individual ACE-score diagnosis. Content must be culturally responsive, disability accessible, and linked to support pathways.

Developmental literacy should be understood as a distributed capacity rather than a curriculum owned by the child. Children need language and concepts, but caregivers, teachers, clinicians, and services must also know how to interpret, receive, and respond. Similarly, epistemic dignity is not the claim that children have adult-like knowledge or full autonomy at all ages. It is the claim that children are developmentally situated knowers whose bodily, emotional, relational, play-based, behavioral, or assisted-communication signals can carry important information about safety, distress, and unmet needs (34–38).

Relational safety is the implementation condition that makes developmental literacy and epistemic dignity usable. Information can become harmful if delivered in ways that shame children, blame caregivers, trigger fear of punishment, overburden teachers, or invite disclosure without support. The interpersonal view of empathy developed by Frederickson is useful here only as a secondary design principle: intervention quality is not defined by benevolent intent or technically correct content alone, but by how a specific child, caregiver, or professional is likely to experience the timing, language, proximity, and challenge of the intervention (53–55).

## 5 Developmental literacy as neurobiopsychosocial public-health education

A central implication of the framework is that neurobiopsychosocial content should be incorporated into age-appropriate early-childhood and school health education where local curricula, safeguarding capacity, and service pathways allow. This does not require a new clinical subject or universal trauma lessons. It requires developmentally staged foundational knowledge about bodies, emotions, stress, sleep, nutrition, relationships, play, care, rights, safety, violence, consent, boundaries, and help-seeking. International public-health and education frameworks already support this direction: health-promoting schools, school health services, the Whole School, Whole Community, Whole Child model, social and emotional learning, mental-health literacy, sexuality education, and violence-prevention guidance all locate health, safety, emotional development, nutrition, relationships, and help-seeking within educational and community systems (20–25, 28–33).

For young children, developmental literacy should be concrete, embodied, and relational. It can include naming feelings and body states, recognizing comfort and discomfort, knowing that children may say no to unwanted touch, identifying safe adults, using play and stories to practice help-seeking, learning that being hit, humiliated, threatened, or mocked is not an acceptable form of care, and understanding basic routines that support health such as sleep, movement, food, comfort, and repair after conflict. The pedagogical aim is not to make children diagnose their families; it is to give children usable concepts and trusted pathways when something feels frightening, confusing, painful, or unsafe (20, 31–33).

For school-age children and adolescents, developmental literacy can expand toward stress physiology, psychosomatic signals, shame, peer dynamics, coercion, digital safety, nutrition, substance-use risk, consent, family conflict, mental health, service navigation, and relational responsibility. Adolescents should also learn that caregiving knowledge is a broadly relevant relational capacity across possible future roles—as parents, co-parents, relatives, professionals, peers, or community members. This is not “parenting readiness” as a fixed trait or a normative pathway into parenthood. It is reflective caregiving knowledge, relational responsibility, and awareness of support needs across diverse life courses.

This content is especially important because family environments are often experienced by children as normal from the inside. Without external concepts, children may interpret violence, neglect, humiliation, emotional unavailability, adult dysregulation, or punitive discipline as deserved, private, ordinary, or impossible to name. Developmental literacy can provide a non-stigmatizing counter-horizon: children learn that feelings and bodily alarm are meaningful; that harmful adult behavior is not the child’s fault; that care includes protection, repair, comfort, and respect; and that specific trusted adults and services exist.

## 6 Epistemic dignity, normalization, and help-seeking

Epistemic dignity addresses a prevention mechanism that is often underdeveloped in child-protection frameworks: children may lack the concepts, credibility, and institutional audience needed to make sense of harm. Epistemic injustice occurs when a person is wronged in their capacity as a knower, either because their testimony is discounted or because the interpretive resources needed to understand their experience are unavailable (36–38). Children are particularly vulnerable to these forms of injustice because they depend on adults for language, safety, interpretation, and institutional access.

The concept is developmentally specific. Infants and toddlers do not provide verbal testimony, yet they communicate through gaze, crying, avoidance, play, sleep, feeding, motor tension, sensory overload, withdrawal, or clinging. Disabled, neurodivergent, and communication-diverse children may communicate distress through behavior, augmentative communication, gesture, routines, shutdown, self-protective avoidance, or caregiver-mediated signals. Epistemic dignity means that these signals are not dismissed as manipulation, misbehavior, attention-seeking, lack of intelligence, or diagnostic noise. It requires responsible hearers who consider context, power, disability, culture, and relational history.

For older children and adolescents, epistemic dignity includes the ability to name experience and expect a non-shaming response. Children exposed to family violence, divorce conflict, humiliation, or emotional dysregulation may engage in threat appraisal, self-blame, loyalty-based silence, or attempts to preserve an idealized image of caregivers (39). Developmental literacy can reduce hermeneutical isolation by giving children language for fear, coercion, shame, boundaries, bodily alarm, and safe help. Epistemic dignity then requires institutions to receive that language without ridicule, disbelief, minimization, or retaliation.

Thus, epistemic dignity is not merely ‘listening to children’ as a procedural gesture. It has three public-health components: children need concepts for interpreting their experience; adults need practices for receiving children’s signals responsibly; and institutions need realistic pathways for support. These components help children become epistemic agents in a developmentally appropriate sense: not autonomous adults, but persons whose emerging understanding can change what adults and systems notice, believe, and do.

## 7 Developmentally staged and disability-inclusive delivery architecture

Developmental specificity is essential. A framework that works mainly for adolescents would not be a child-health framework. Table 2 therefore describes a staged delivery architecture from infancy to transition into adulthood. The earliest levels are caregiver-mediated, sensory-aware, and play-based; the later levels become increasingly verbal, reflective, rights-based, and service-navigation oriented. Across all stages, children are not made responsible for solving conditions they cannot control. Adults and institutions remain responsible for safety, response, and support.

**Table 2:** Developmentally staged and disability-inclusive delivery architecture.

Developmental period	Primary developmental focus	Core developmental-literacy content	Primary delivery settings	Safeguards
Infancy (0–12 months)	Safety, sensory regulation, feeding, sleep, co-regulation, caregiver attunement.	Caregiver recognition of cues, soothing, responsive caregiving, caregiver mental health, nutrition, safe environments.	Pediatric care, home visiting, early intervention, family services, community health.	Do not locate responsibility in the infant; support the relational environment and caregiver capacity.
Toddlerhood (1–3 years)	Autonomy, play, early language, affect regulation, sensory needs, attachment exploration.	Naming body states and feelings through routines and play; safe touch; comfort; caregiver-mediated help-seeking.	Early childhood education and care, pediatric visits, early intervention, family support.	Avoid pathologizing behavior; interpret distress within development, sensory needs, communication, and context.
Preschool (3–5 years)	Symbolic play, simple rules, peer interaction, early self-advocacy.	Feelings, body boundaries, private parts, safe and unsafe situations, trusted adults, repair after conflict, comfort, and basic health routines.	Kindergarten/preschool, ECEC, family centers, community health.	Use visual, story-based, play-based, multilingual, AAC-compatible, and sensory-aware materials.
School age (6–11 years)	Belonging, learning, peer relations, moral rules, concrete understanding of rights and safety.	Stress and body signals, nutrition, sleep, bullying, violence, neglect, shame, safe adults, rights, and how and where to seek help.	Primary schools, school health services, pediatric/public-health touchpoints, community programs.	Do not invite unsupported disclosure; pair learning with clear confidentiality boundaries and referral routes.
Early adolescence (12–15 years)	Identity, puberty, autonomy, peer norms, digital life, emotional complexity.	Mental-health literacy, stress physiology, consent, coercion, relationships, digital safety, substance-use risk, support systems.	Secondary schools, youth services, primary care, digital and community services.	Avoid over-pathologizing normal distress; distinguish literacy and help-seeking from therapy.
Later adolescence and transition (16–18 + years)	Future roles, intimacy, civic responsibility, transition to adult services.	Reflective caregiving knowledge across possible roles, co-parenting awareness, service navigation, relational responsibility, support needs, and non-coercive help-seeking.	Secondary/vocational schools, reproductive health, youth and family services, community programs.	Never infer future caregiving capacity from diagnosis, disability, adversity history, poverty, or family background.

The framework is also disability-inclusive by design. Disability and neurodivergence are not peripheral exceptions. They are design conditions. Developmental literacy must not treat typical development as a hidden norm against which children, caregivers, or families are judged. Materials should be predictable, multimodal, sensory-aware, linguistically accessible, culturally responsive, compatible with augmentative and alternative communication, and adaptable to early intervention and ECSE contexts. Co-design should include disabled and neurodivergent children, caregivers, disability advocates, educators, early-intervention professionals, and families whose experiences are often shaped by institutional misinterpretation or exclusion (40–43, 57).

An anti-ableist and anti-bias prevention model also avoids using diagnosis, communication style, school achievement, behavior labels, poverty, migration status, family structure, neurodivergence, disability, or prior adversity as proxies for parenting capacity or child risk. It focuses instead on dynamic support needs, relational safety, service accessibility, and modifiable developmental environments. Parenting and caregiving capacity are not inferred from group membership; they are supported through voluntary, accessible, relationally safe services.

## 8 Implementation strategy and safeguards

Implementation should follow proportionate universalism: universal access to developmental knowledge and safe help-seeking, combined with greater support as social, relational, and developmental need increases (50). Schools are important because they provide near-universal reach, shared language, and repeated contact. However, the framework is not school-only. It requires coordination among early-childhood services, health-promoting schools, pediatric care, public health, family support, social services, youth services, disability services, and community systems (20–25).

The Icelandic Prevention Model illustrates the relevant public-health logic. Its value for this manuscript is not individual trauma detection, but data-informed environmental prevention: repeated population-level assessment, local coalitions, norm change, parental and community engagement, and modification of the social conditions that shape youth risk and protection (51). A similar logic can inform developmental literacy: monitor environments and service pathways, then change conditions, not merely identify vulnerable individuals.

Trauma-informed language should be used cautiously. Trauma-informed practice is not inherently harmless, and adversity does not inevitably result in pathology. School and early-childhood systems should not make teachers “trauma detectives,” invite uncontained disclosure, or frame children through deficit narratives. Critical work in early childhood education also warns that systems can reproduce harm through exclusion, surveillance, ableist interpretation, or biased responses to behavior. The safer public-health strategy is to provide non-stigmatizing knowledge, relationally safe interaction, clear confidentiality boundaries, accessible referral maps, staff supervision, anti-bias practice, and monitoring of unintended harms such as shame, self-surveillance, disclosure without support, teacher burden, excessive pathologization, biased interpretation, and institutional mistrust (44–46, 56).

Table 3 summarizes implementation risks and safeguards. The central rule is that developmental literacy must always be paired with response capacity. A child who learns that violence is wrong but has no trusted adult, confidential route, or accountable service pathway may become more isolated, not safer. Similarly, a teacher who receives disclosure without training, time, protocols, supervision, or referral capacity is placed in an ethically unsafe position. Implementation therefore requires governance, training, referral infrastructure, data protection, anti-bias practice, co-design, and outcome evaluation.

**Table 3:** Implementation risks and safeguards.

Implementation risk	Safeguard	Evaluation indicator
ACE determinism and individual prediction	Use ACEs as population-level signals; avoid ACE scores as individual diagnosis or school-based risk labels.	Language audit; absence of ACE-score-based classification; monitoring for stigma and false reassurance.
Trauma-informed overreach	Treat trauma-informed practice as an implementation hypothesis, not an inherently harmless approach; avoid trauma-detective practices, unsupported disclosure, and trauma as a default explanation for all behavior.	Adverse-response monitoring; staff supervision; child/caregiver feedback; teacher burden and iatrogenic-harm checks.
Ableism and deficit framing	Apply Universal Design, ECSE expertise, disability co-design, multimodal communication, anti-ableist language, and explicit safeguards against using diagnosis or behavior labels as risk proxies.	Accessibility testing; disabled/neurodivergent participant feedback; equity audit; documentation of adaptations and response patterns.
Schools becoming clinics or surveillance sites	Keep schools as health-promoting and help-seeking platforms; separate education from clinical diagnosis and statutory investigation.	Role clarity; teacher workload; referral completion; consent and confidentiality procedures.
Disclosure without support	Teach help-seeking only where referral maps, safeguarding protocols, supervision, and service access exist.	Time to response; referral completion; child-rated safety after disclosure.
Caregiver blame	Frame caregiving as learnable and support-dependent; include caregiver mental health, poverty, stress, and service access.	Caregiver acceptability; shame/stigma scales; engagement with voluntary supports.
Data misuse and privacy risk	Use data minimization, clear governance, confidentiality limits, and ethical review for any structured identification.	Privacy audit; complaint monitoring; documentation of data flows and safeguards.
Cultural mismatch	Co-design and adapt materials with local families, youth, educators, disability advocates, and services.	Cultural acceptability; qualitative feedback; adaptation fidelity and local ownership.
Biased adult interpretation of children’s signals	Use anti-bias, disability-informed, and culture-sensitive training; examine whether distress is dismissed or ridiculed because of behavior labels, disability, gender, race, class, family form, migration status, or communication style.	Equity audit; differential response patterns; vignette-based staff assessment; child/caregiver feedback; review of dismissal or ridicule incidents.

## 9 Negative intervention analysis: boundary of the framework

The framework includes a negative intervention analysis only to clarify its boundary and should not be read as a reproductive-policy paper. Coercive reproductive control may appear superficially preventive because it intervenes before possible future harm. However, it operates at the wrong causal level. It targets reproductive status or group category rather than caregiving conditions, poverty, violence, relational safety, treatment access, social support, service accessibility, and institutional trust (52).

Its predictive logic is weak. Diagnosis, disability, neurodivergence, poverty, educational attainment, previous adversity, or social marginalization are poor stand-ins for actual caregiving quality. Many people with histories of adversity, mental illness, disability, or social exclusion become safe and reflective caregivers when supported; many apparently low-risk adults do not. Group-based reproductive restriction therefore produces false positives, false negatives, stigma, concealment, and avoidance of help.

This point is not a claim about who should parent. It is an argument about public-health mechanism. Humane prevention is reversible, support-oriented, adaptive, relational, rights-compatible, and targeted to modifiable developmental environments. Coercive reproductive control is low-precision, stigmatizing, rights-violating, and corrosive of the trust on which early help-seeking depends. The rejected comparator therefore reinforces the positive framework: primary child protection should build knowledge, relationships, support, and safe institutions, not classify people as reproductively worthy or unworthy.

## 10 Evaluation framework and testable hypotheses

Because this article proposes a Hypothesis and Theory framework, its value should be judged by the clarity and testability of the questions it generates. Evaluation should examine child, caregiver, professional, school, service, and system-level outcomes. Designs may include feasibility studies, co-design studies, cluster pilots, stepped-wedge implementation trials, mixed-methods process evaluations, and longitudinal population monitoring. Implementation outcomes should include reach, acceptability, appropriateness, feasibility, fidelity, cost, penetration, sustainability, equity, response quality, and referral completion (47–49).

Table 4 translates the framework into testable hypotheses. The hypotheses are not claims that developmental literacy alone prevents ACEs. They specify intermediate mechanisms that are empirically assessable: knowledge, recognition, help-seeking, trust, relational safety, disability accessibility, caregiver reflective capacity, response quality, service linkage, and unintended harms. Long-term ACE reduction would require larger, multi-level studies and should be evaluated cautiously.

**Table 4:** Testable hypotheses and evaluation indicators.

Hypothesis	Level	Primary outcomes	Illustrative indicators/designs
H1. Developmentally staged materials improve knowledge about bodies, emotions, stress, care, safety, rights, and help-seeking compared with age-generic materials.	Child/adolescent	Developmental knowledge and recognition accuracy.	Pre/post knowledge items; vignette tasks; age-specific comprehension testing.
H2. Epistemic-dignity practices increase children’s perceived credibility, ability to name distress, and confidence in approaching trusted adults.	Child/adolescent and institutional	Perceived credibility, naming capacity, help-seeking confidence.	Child-friendly surveys; qualitative interviews; disclosure experience audits.
H3. Caregiver-mediated early-childhood delivery improves caregiver cue interpretation, co-regulation confidence, and responsive caregiving.	Early childhood and caregiver	Caregiver reflective capacity, interaction quality, stress regulation.	Caregiver-report scales; observed interaction; early-intervention feasibility studies.
H4. Relationally safe delivery reduces shame and increases trust in educators, clinicians, and services.	Child, caregiver, professional	Trust, stigma, perceived coercion, service engagement.	Trust/stigma scales; qualitative process evaluation; adverse-response monitoring.
H5. Disability-accessible and co-designed materials improve comprehension, acceptability, and usability for disabled and neurodivergent children, caregivers, and professionals.	Equity and accessibility	Accessibility, usability, participation equity, and response credibility.	UDL/accessibility audit; subgroup analyses; co-design feedback; AAC and sensory-accessibility testing.
H6. Integrated school, health, caregiver, and community delivery produces higher reach and referral completion than single-setting approaches.	Systems	Reach, continuity, uptake, referral completion.	Cluster pilots; stepped-wedge designs; implementation outcomes.
H7. Programs that monitor unintended harms show higher acceptability and safer implementation than programs measuring knowledge gains alone.	Implementation safety	Acceptability, appropriateness, adverse responses, teacher burden.	Mixed-methods implementation studies; harm-monitoring dashboards.
H8. Upstream developmental prevention reduces support for coercive reproductive control and increases support for voluntary, rights-compatible, environmental prevention.	Policy attitudes	Support for non-coercive prevention and service investment.	Policy-attitude items; community evaluations; pre/post follow-up.
H9. Anti-bias and disability-informed response protocols reduce dismissal, ridicule, and biased interpretation of children’s distress signals compared with information-only implementation.	Institutional/professional	Response quality, credibility, equity, and child-rated safety.	Staff vignette tasks; response-quality audits; child/caregiver feedback; differential referral and follow-up patterns.

Measurement development is a central next step. Developmental literacy could be assessed through age-appropriate knowledge items, vignette tasks, body-signal recognition, help-seeking maps, and caregiver/professional knowledge. Epistemic dignity could be assessed through perceived credibility, child-friendly participation, response quality, disclosure experience, and qualitative accounts of whether children felt taken seriously. Relational safety could be assessed through trust, shame, perceived coercion, confidentiality understanding, and user experience. Disability accessibility should be measured directly rather than assumed.

## 11 Discussion

The main contribution of this manuscript is integrative. It connects ACE/PCE research, toxic stress, relational health, nurturing care, school health, child rights, epistemic injustice, disability-inclusive implementation, anti-bias practice, and implementation science into one upstream public-health framework for primary child protection. The concepts of developmental literacy and epistemic dignity provide bridging terms for a recurring problem: children, caregivers, educators, clinicians, and institutions often lack shared language for recognizing and responding to early relational and psychobiological risk before crisis becomes the entry point to care.

A second contribution is to reposition school-based prevention. Schools are not presented as universal solutions, clinical substitutes, or screening sites for ACE-score classification. They are one component of a wider child-health system that can provide shared developmental language, violence-prevention norms, health education, relational safety, and help-seeking routes when linked to families, health services, safeguarding infrastructure, and community supports. This framing aligns with health-promoting schools and avoids overburdening educators with clinical or statutory functions for which they may not be resourced (22–25, 44–46).

A third contribution is to deepen child participation through epistemic dignity. Children are not merely recipients of protection. They are developmentally situated knowers whose signals, interpretations, and emerging agency matter. For preverbal, disabled, neurodivergent, and communication-diverse children, this requires a broad view of communication and a responsible-hearer model. For older children and adolescents, it requires concepts, credibility, and pathways that make help-seeking possible without shame or retaliation.

The framework has limitations. It is a theory-generating synthesis rather than a systematic review or empirical trial. Developmental literacy and epistemic dignity require measurement development, cross-cultural adaptation, disability-community co-design, and evaluation in real service systems. Concepts of care, family authority, school authority, privacy, disclosure, corporal punishment, disability, and help-seeking vary across settings. The model cannot compensate for structural adversity, poverty, violence, service shortages, or weak safeguarding systems. Finally, the negative intervention analysis must remain subordinate to the positive child-health framework; otherwise, the manuscript could be misread as centered on reproductive policy rather than primary child protection.

Despite these limitations, the proposed model is intentionally testable. It does not claim that information alone prevents ACEs. It claims that developmental knowledge, epistemic recognition, relational safety, caregiver support, and service-linked environments are plausible, modifiable, and evaluable components of upstream child public health.

## 12 Conclusion

Primary child protection should begin before crisis becomes the main route into care. A humane public-health approach should not ask who is worthy of reproduction or which child has a deterministic ACE profile. It should ask how societies can make developmental harm less likely by strengthening children’s concepts, caregivers’ support, educators’ practices, service pathways, and institutional responses.

Developmental literacy and epistemic dignity are proposed as linked constructs for this task. Developmental literacy gives children, caregivers, educators, clinicians, and institutions age-appropriate and disability-accessible knowledge about bodies, feelings, stress, care, nutrition, relationships, boundaries, rights, and help-seeking. Epistemic dignity ensures that children’s signals are taken seriously and translated into support. Together with relational safety, health-promoting schools, nurturing care, caregiver support, and community-linked services, these constructs offer a developmentally staged, disability-inclusive, anti-bias, and non-coercive framework for preventing ACE-related harm.

## Data Availability

The original contributions presented in the study are included in the article/supplementary material, further inquiries can be directed to the corresponding author.
